# A Left-Sided Prevalence of Lentigo Maligna: A UK Based Observational Study and Review of the Evidence

**DOI:** 10.1155/2015/310270

**Published:** 2015-04-01

**Authors:** Mark Gorman, Andrew Hart, Bipin Mathew

**Affiliations:** ^1^Canniesburn Plastic Surgery Unit, Glasgow Royal Infirmary, UK; ^2^Hull Royal Infirmary, Hull, UK

## Abstract

Skin cancer has been shown to present asymmetrically, prevalent on the left side of the body, more so in subtypes of cutaneous melanoma such as lentigo maligna. Biases have been linked to cumulative UV light exposure and automobile driving patterns. Though left-right ratios have previously correlated with the side men or women tend to position themselves or countries drive on, more recent trends indicate a consistent left-sided bias. To clarify reasons for changing trends, a review of the evidence base and LM's laterality in a UK cohort (99 cases 2000–2011) was conducted for the first time. The strong correlation of left-sided excess, found in both genders (ratios 1.381–1.5, *P* < 0.05  *X* ^2^ 0.841), is congruent with more recent findings. Though evidence indicates that driving position is no longer a risk factor for LM, due most likely to improved car window UV protection, it remains the most commonly attributed cause. Understanding phenomena such as UV lights “scatter effect” or that cumulative exposure may not be a significant risk factor helps rationalize older conclusions that would otherwise appear contradictory. The reasons for left-sided excess remain unclear but may be due to factors requiring further research such as the body's anatomical/embryological asymmetry.

## 1. Introduction

Since the late 1970s, studies have shown trends in which cancer presents with a bias to one side of the body [1, 2]. This has been most commonly reported in breast cancer, where a consistent left-sided excess has been shown [3]. Over the last decade, studies have also started to evidence a consistent trend of skin cancer on the left side of the body [4–6]. This work has focused more on invasive melanoma and nonmelanoma skin cancer (NMSC) than melanoma in situ (MIS) or lentigo maligna (LM). When data relating to LM has been reviewed, not only do the biases in laterality remain, but interestingly, the proportional shift seems to be more profound [7, 8]. These findings have been most commonly attributed to environmental factors such as sun damage, where it is speculated that patterns observed may relate to driving behaviors, such as positioning in a car and the resulting ultraviolet (UV) light exposure [5]. Laterality biases on the left and right sides of the body have therefore been previously linked to which side of the road individuals drive on [8–10], but more recent work indicates a left-sided bias irrespective of such variables [4, 7]. Despite changing trends, there has been little critique of this body of work thus far. This study investigates LM's laterality in a UK cohort and reviews the evidence base to date.

As highlighted in our previous work [11], LM presents a challenge to pathologists in terms of its histological diagnosis as it can be difficult to confidently decide whether excision margins are clear. This is especially important when making management decision, such as whether to further excise lesions that present predominantly on cosmetically sensitive areas in the head and neck region [12]. Given that this is a difficult pathology to manage, it is important to identify factors that may reduce LM's risk.

Driving pattern's association with sidedness of LM at diagnosis has been found to vary based upon gender. In an Australian cohort of patients (1984–1990), the prevalence of LM was higher on the right side for men and left-side for women, explained by driving habits: men more commonly situated in the driving seat (right side) and women on the opposite passenger side [8]. However, more recent epidemiological work, with data from six different populations (including the UK), has found a consistent excess of left-sided cutaneous melanomas, irrespective of gender and driver side [4]. This has been supported by data related to MIS [7] and LM within US populations [6]. To date there have been no UK based studies investigating LM's intersex or anatomical left to right ratio.

Cumulative sunlight has been long assumed to increase melanoma risk, with a stronger association demonstrated in LM, based upon its higher preponderance in the elderly and presentation on areas of direct exposure [12]. However, case control studies over the last two decades challenge this assumption, showing that risk is increased by intermittent periods of UV exposure resulting in skin sun damage, as opposed to the total hours of sunlight [13–15]. Despite such findings, the importance of intermittent exposure has to date received little research focus, and the “cumulative hypothesis” persists in most causative explanations.

Intermittent, as opposed to cumulative, UV light would seem to explain why LM has previously been shown to present more prevalently on the side of maximum exposure whilst in a car. It does not explain why this pattern has changed, such that, irrespective of individuals positioning within a car or which side of the road they drive on, trends now indicate a consistent left-sided excess. In light of these findings, we have revisited the question of LM laterality within a UK cohort of drivers seated on the right side (affording comparison with Foley et al.'s previous cohort).

## 2. Methods

Patient consent was not required and the experimental methods observed the ethical principles of the Declaration of Helsinki.

### 2.1. Laterality Study

As a retrospective review (as part of the aforementioned wider trial), 99 cases of LM (2000–2011) were identified from a UK histopathology department (Hull and East Yorkshire NHS Trust) [11]. Cases were excluded if reports indicated invasive melanoma (LMM) or required information was omitted. The anatomical site, side, and patient demographics (gender and age) were all recorded from individual's histopathology reports.

### 2.2. Review of the Laterality Evidence Base

A review of the laterality evidence base to date was conducted within “Papers 2” (search engine and reference managing software available for mac and pc) [16]. “Papers 2” allows the user to define the domains (single or multiple, such as “title,” “abstract,” “general,” “year,” and “author”) and portals (we selected PubMed and Google scholar), to better stratify how the chosen search terms identify relevant articles/sources of evidence. Searches were thus conducted looking within “titles” and “general,” using the search terms “*melanoma, lentigo, cutaneous, skin, laterality, side, left, right and cancer.*” Abstracts were screened and articles relating to cutaneous melanoma/NMSC and LM laterality were selected for review (13 in all, five with data pertaining to MIS/LM laterality and one study exclusively focused on LM). Articles were then selected regarding the relationship between laterality, cancer in general, and embryology/epidemiological risk factors (15 articles). Due to the small pool of relevant papers, though higher levels of evidence were sought, no exclusion criteria were required.

### 2.3. Statistics

The majority of previous laterality studies had employed* X* ^2^ and odds ratio in their analysis. For direct comparison, we have employed the same statistical tests.

Pearson's* X* ^2^ test was used to analyze the probability of independence (*P* < 0.05) regarding the pattern of laterality observed between genders in the presentation of LM in the head and neck region (as in Foley et al.'s previous study) [8]. If the *X* ^2^ value was found to be less than 3.841 [17], the null hypothesis should be rejected, and males and females would be considered to follow the same pattern of laterality (df = 1) [18]. The left-right laterality odds ratio (or strictly just the ratio) was then calculated for both genders.

## 3. Results

LM was more common on the left side of the body (59%, *n* = 58) than on the right one (42%, *n* = 41, Table 1). This was true for both genders, the left-sided male prevalence being 58% and the female one being 60%. The left-right ratios for men, 1.381, and women, 1.5, both indicate a very strong association of left-sided excess.

The odds ratio (0.9206) and *X* ^2^ (0.841 = reject null hypothesis *P* < 0.05) indicate that there was no significant difference in laterality patterns between genders.

Of the 99 cases, the majority of lesions presented in the head and neck region (*n* = 95/>95%, Table 1) and in the elderly, in those over 55 years of age (97%, Table 2).

## 4. Discussion

The strong correlation (1.381 and 1.5, resp.) in men and women towards a left-sided excess (~60%) and significant homogeneity (*P* < 0.05* X* ^2^ 0.841) between genders indicate a different pattern of LM distribution than was found in older studies [8–10]. Foley et al. found both an intersex variability and a preponderance of a right-sided prevalence in those more commonly positioned on the right side of the car. Our findings are congruent with more recent trends that indicate a consistent left-sided bias [19]. In our UK cohort, the proportional shift in laterality bias seems to be more profound than when compared with cutaneous melanoma in general, supporting previous data from the US and Australia [7, 8].

Returning first to the issue of gender based laterality variation, Foley et al. demonstrated a female left-sided and male a right-sided bias [20]. This was attributed to women more commonly occupying the passengers' seat. Foley et al. explained that their findings occurred in the pre-air conditioning era, leading to driving with windows down. Thus, the protection that may be afforded by glass (filtering 90% of the UV-B sunlight) was lost. Accepting this causation, our findings may, in a historical sense, follow those of Foley et al. in that our UK cohort drove approximately 20 years later, when air conditioning had become the norm and the speculated preponderance of men more commonly being on the driver's side had all but disappeared [21]. On further scrutiny, however, though Foley et al.'s conclusions may be partly valid, they are contradicted by some of their own later work and cannot be used to explain why cutaneous melanoma now seems to have a consistent left-sided bias irrespective of the causations they cite.

Continuing associative links between asymmetrical presentation and driving habits have persisted mainly in US based studies involving left-seated drivers [5, 7]. Such assertions are contradicted by both the findings of this study and the epidemiological analysis of national cancer registries, including countries where drivers are positioned/seated on the right (i.e., England, Scotland, and Australia), and the left-sided excess of cutaneous melanomas remains [19]. The left-right ratio across the six counties sampled was found to be consistently greater than 1.10 (range 1.08–1.18), the collective cohort totaling just under 100,000 cases. Given the size of the overall sample and that the ratio remained very similar across countries, it is unlikely that that trend is simply due to chance. Reflecting on their work, Brewster et al. rule out more straightforward explanations, such as recording bias and handedness of sunscreen application. Unlike previous findings, no significant differences were observed between or within subgroups, based upon gender, age, or anatomical site (i.e., upper versus lower limb, not laterality).

Factors leading to greater levels of left-sided sun exposure, other than positioning in a car, would seem to offer the most obvious explanation for laterality biases, but to date, no plausible hypotheses have been suggested. Given that countries from both the north and south hemispheres indicate the same trend, differences such as the direction of sun set/rise and sunbathing habits may be ruled out. There has been little work investigating the link between melanoma and varying sun patterns and none that relates data directly to the laterality of presentation. On review, the only related UK melanoma study to demonstrate such data includes no analysis of laterality [22]. Though driving patterns do not explain the more recent trends, Brewster and de Vries [4] believe that they may still be a risk factor for NMSC. Without plausible epidemiological links to explain a left-sided preponderance of cutaneous melanoma, alternative explanations have been sought and considered.

An interesting hypothesis is that of the body's asymmetry. In a UK study looking at cancer in five major paired organs (the breasts, lungs, kidneys, testes, and ovaries, including ~250,000 patients), it was found that organ asymmetries, as opposed to epidemiological factors, coincided closely with the laterality of presentation [23]. Relating more specifically to melanoma, it has been cited that asymmetry of the lymphatic and circulatory systems may influence the immune response against or patterns of melanoma spread [19]. As yet, there has been little specific research with which to support such ideas. Brewster et al. cite a recent study involving the signaling molecule “Nodal” (belonging to the TGF-b superfamily) [24, 25], which is both involved in the left-right ratio of embryogenesis and histopathologically present when melanoma tissue excisions have been examined, not seen in normal skin. Brewster et al. hypothesize a link between these findings and data trends within the national cancer registries from their epidemiological study. Further analysis of US data (SEER “White” registry 1998–2003) revealed a more profound excess of left-sided tumors with distant metastases (ratio 1.19), compared with localized/regional spread tumors (ratios 1.09/1.06, resp.), suggesting that, as “Nodal” is secreted in more aggressive melanomas, it may be influencing the left-right ratio pattern. Individuals with situs inversus may provide the perfect cohort with which to investigate anatomical/embryological asymmetry. That no research has yet been conducted will owe in part to its rare incidence (estimated at ~1 : 8000–25000) [26].

There are few other studies demonstrating the same right-sided tendency found in Foley et al.'s study, and the evidence that does exist relates to particular subgroup stratifications of older individuals. The pattern has been demonstrated in two studies of NMSC and one of melanoma. Two are US based, the first involving a cohort from Latino/Hispanic descent presenting with NMSC [27]. The second showed an increased prevalence of right-sided melanoma in women presenting on specific anatomical sites, namely, MIS of the ear/trunk and invasive melanoma of the eyelid [6]. In both studies, all other subgroups were found to have a small left-sided majority. In the first study, it was theorized that more passengers travelled on the passenger (right) side due to decreased car ownership and increased members per Latino/Hispanic household. However, this was merely an extrapolative inference from current population statistics, and it was not based upon direct observation or data from the correct era. For the second study, the same conclusions and intersex variation apply as demonstrated in Foley et al.'s 1980s Australian cohort, when women historically occupied the passenger seat more commonly.

The other study to evidence a right-sided laterality bias was also undertaken by Foley et al. Preceding their LM study, they investigated patterns of solar keratosis (SK) presentation (related again to driving), aiming to test anecdotal assertions of the time, that skin cancer was “more frequent on the right side in Australia and Britain” ([9] page 18). They found a similar general pattern of intersex variation as within their later LM study, with more right-sided male lesions and vice versa for women. Importantly, though, this time it occurred only on the right arm for men and the head and neck region for women. Subsequent papers quote Foley's studies together as evidence to support the “window down exposure theory.” However, when doing so, authors have failed to address that the studies' conclusions were somewhat contradictory. It was argued that, by being taller, men had fewer head and neck SKs, protected by the shade of car roofs. This line of argument was then omitted, despite circumstances being the same, when explaining the anatomical distribution of the LM data, for which both genders had an overwhelming majority of head and neck lesions. A US study carried out during the same period with similar driving conditions found no apparent increase in photo damage with windows down (panel based assessment of photos) but still found that asymmetry correlated with time spent driving seated on the left [10].

Paulson et al. [28] highlight that if driving habit associated UV patterns do influence the left-sided excess observed with melanoma, there would seem to be a disproportionately large number of lesions presenting on areas rarely exposed whilst in a car, such as the legs. Similarly, though the vast majority of LM lesions present in the head and neck region (>95% in our study), Foley et al.'s aforementioned contradictory conclusions also indicated that men's high prevalence of head and neck lesions occurred in spite of protective shade. The often cited “window down exposure theory” would seem to require more adequate explanation and may be answered by UV radiation's “scatter effect.” It is estimated that 50% of UV-A radiation is received despite shielding from direct sunlight, be it umbrella shade or car roof. Thus, areas that are not in direct line of visible light are still affected, but prevalence should increase the nearer a body part is to an unprotected portal of entry (i.e., passenger window) [29]. The historical improvement in UV protection levels afforded by car windows, previously almost nonexistent, explains why in Singer et al.'s older study it was found that having “windows down” did not increase the positive photo damage correlation observed with driving hours in general [30]. Though the degree to which modern cars guard against UV-A (as opposed to UV-B) has been questioned [31], improvements seem the most plausible explanation as to why laterality no longer matches with driving side. Follow-up studies looking at direct skin photo damage and not just skin cancer prevalence would be useful in order to clarify this point. A summarized review of the discussion's learning points and study findings are presented as follows.

*Key Findings and Learning Points from This Study*. This is the first study to evidence significant* left-sided excess/prevalence of LM in a UK based population*.

*(i) Previous Evidence* linked laterality bias to car related UV light exposure; its key examples are as follows:

- LM prevalence was significantly higher on the* right side for men* and left side for women (in both the head and neck region) explained by driving habits,* men more commonly situated in the driving seat (right side)* and women on the opposite passenger's side [8];
- SKs and NMSC most commonly presented on the arm of the driver's side [9].

*(ii) Recent Trends* indicate that cutaneous melanoma prevalence now has consistent* left-sided excess*, not affected by gender or which side of the road countries drive [4, 6, 7, 19]:

- combined with our study, findings of the last decade indicate driving habits are no longer a risk factor for LM/melanoma (see below points);
- a shift to left-sided excess is explained by (a)* the previous lack of UV protection afforded by car windows* +/− (b)* driving with windows down* (which stopped post the advent of air conditioning).

*(iii) Attribution Error*. Despite evidence indicating that driving position is no longer an associated risk factor, authors still commonly cite it as the most plausible cause. Such causation error/prior assumptions may be refined with the knowledge of the following.

- UV radiation's “Scatter Effect”: 50% of UV-A radiation is received despite shielding from direct sunlight, for example, a car roof or umbrella shade. Thus, areas not in the direct line of visible light are still affected and it is the relationship of UV intensity versus filtration, not shade, which is key [29].
- The evidence points to the dose of UV radiation passing through newer car windows as posing no risk, and though counterintuitive, such chronic low grade UV exposure may even be protective against melanoma (and LM) [14, 15, 32].

*(iv) Moving Forward*

- Research trends indicate that that there is no evidence justifying the need for additional sun cream protection whilst in a car (due to improved UV window protection) cited by many recent studies.
- The left-sided excess of cutaneous melanoma requires further research, which may, for example, include study of the body's anatomical asymmetry or epidemiological factors other than driving.
- Identifying risk factors/causation in LM/LMM is even more important as compared with other subtypes of melanoma; it is on the rise and demonstrates a more profound shift towards left-sided excess.

A point more widely discussed, but not within previous laterality studies, is that while LM occurs over severely sun damaged skin (part of its diagnostic histopathological criteria) [32, 33], it should not be confused with the potentiating effects of chronic sun exposure, which, without resultant “damage” per se, has been shown to reduce melanoma risk, assessed through occupational exposure groups that work outdoors [13–15, 32]. Though counterintuitive, low dose UV radiation passing through newer car windows may have very little, no, or even mildly protective effect regarding LM's prevalence and laterality of presentation. The conclusions of many recent studies that people should be more vigilant regarding sun protection whilst in cars may thus be overstated [27, 28].

## 5. Conclusion

This is first UK based study investigating LM's laterality, where the left-sided excess found was the same for both sexes, contrasting with older findings. Changing trends in cutaneous melanoma's laterality may be explained by factors such as the lack of protection previously afforded by car windows. Despite evidence indicating that driving position is no longer an associated risk factor for LM or cutaneous melanoma, it remains the most commonly attributed cause: an “attribution error.” It is important to scrutinize the causation of LM's left-sided excess, as found by this and more recent studies, towards the identification of more likely factors, be they epidemiological* or* based upon further study of the body's anatomical/embryological asymmetry. Understanding that cumulative UV exposure may not be a significant risk factor, and being aware of UV lights “scatter effect” phenomena, may help direct future research efforts and rationalize older findings.

## Figures and Tables

**Table 1:** Variance in LM lesions anatomical site presentation for side and gender distribution.

Anatomical & intersex variation	Site distribution	
	Left side	Right side
Head & neck	56 (58%)	39 (42%)
Men	29 (58%)	21 (42%)
Women	27 (60%)	18 (40%)
Limbs	2 (50%)	2 (50%)

Total	58 (59 %)	41 (41%)

**Table 2:** Demographics of patients presenting with LM.

Gender	
*N* (total)	99
Male	52 (52%)
Female	47 (47%)
Age (years)	
Median (IQR)	72 (64, 79)
[Range]	[35, 100]
<55	12 (12%)
55–74	45 (45%)
75+	42 (42%)
